# Hæmorrhage after Extraction of Teeth

**Published:** 1868-12

**Authors:** 


*Hæmorrhage after Extraction of Teeth.*—A correspondent of the *London Lancet* recommends the following treatment for alveolar hæmorrhage: "Soften a bit of white wax, and mould it into a conical shape about an inch long, and press it into the cavity, at first lightly, and then very firmly, so as to fill it. Cover this with a thick pad of lint, to retain it in its place, and bind the jaws together for a few hours with some kind of bandage." While this method is not by any means a new one, we would suggest the cleansing out of the alveolar cavity, from which the tooth has been extracted, as thoroughly as possible prior to introducing the wax.

A much better method than the above, and one also not new, is, in the case of teeth with a single root, and where the root is not broken up in the attempt to remove it, to carefully cleanse it of blood, and after preparing the cavity to coat the root with warm wax and immediately return it to its socket.

Where this is not possible, owing to the tooth having more than one root, or where the root is removed in fragments, an effectual method is to saturate a pellet of cotton with sandarach varnish, on which is taken up Monsel's Powd. Sub. Sulphate of Iron, and, after carefully cleansing the cavity, inserted over the mouth of the bleeding vessel. Soft wax or cotton may be placed on this first pellet, and the cavity be thus filled up. If necessary a compress made from a piece of cork, may be applied over the cotton filling the cavity, and held in place by the opposing teeth when the mouth is closed.

